# Marine heatwaves of different magnitudes have contrasting effects on herbivore behaviour

**DOI:** 10.1038/s41598-022-21567-9

**Published:** 2022-10-15

**Authors:** Patrick W. S. Joyce, Wing Yee Tang, Laura J. Falkenberg

**Affiliations:** Simon F.S. Li Marine Science Laboratory, The Chinese University of Hong Kong, Shatin, New Territories, Hong Kong SAR, China

**Keywords:** Ecophysiology, Behavioural ecology, Climate-change ecology, Tropical ecology, Marine biology

## Abstract

Global climate change is leading to shifts in abiotic conditions. Short-term temperature stresses induced by marine heatwaves (MHWs) can affect organisms both during and after the events. However, the recovery capacity of organisms is likely dependent on the magnitude of the initial stress event. Here, we experimentally assessed the effect of MHW magnitude on behavioural and physiological responses of a common marine gastropod, *Lunella granulata*, both during and after the MHW. Self-righting behaviours tended to become faster under moderate MHWs, whereas there was a trend toward these behaviours slowing under extreme MHWs. After a recovery period at ambient temperatures, individuals that experienced extreme MHWs showed persistent small, but not significant, negative effects. Survival and oxygen consumption rates were unaffected by MHW magnitude both during and after the event. While extreme MHWs may have negative behavioural consequences for tropical marine gastropods, their physiological responses may allow continued survival.

## Introduction

With increasing anthropogenic inputs, global environmental conditions are shifting, thus it is imperative that we understand how organisms and ecosystems will respond to future climate change. Species responses to environmental changes will depend, in part, on the extent to which the stressor is experienced (i.e., the magnitude of change). Stressful environments can lead to mortality of individuals or have sublethal effects on organisms through alteration of behavioural and physiological functioning^1–3^. Moderate increases in temperature can induce positive effects on certain organisms (e.g., increased metabolism in ectotherms) until a critical threshold is reached and the maximum process rate occurs^4,5^. If the stressor exceeds this critical threshold, effects become negative and biological processes may become impaired leading to mortality events^6,7^.

An important stressor in marine systems is temperature, with the role of this component likely to increase as global warming continues. Along with increased gradual warming predicted for the coming decades, marine heatwaves (MHWs)—short term temperature anomalies above the 90th percentile of historic temperatures lasting at least 5 days^8^—are also anticipated to become more frequent, have greater intensity, and last longer^9,10^. With their increasingly prominent occurrence in ecosystems, more research is required to understand the biological and physiological effects of MHWs. Severe MHWs are recognised to have immediate, large-scale effects by driving increases in mortality of foundation species^11,12^. Yet, potential exists that short-term MHWs could have comparable impacts were they to induce negative impacts on behaviour or physiological functioning; a pattern which is coming to be recognised in an increasing range of organisms^13,14^.

As MHWs act on ecosystems over relatively short timescales (cf. gradual long-term warming), their impacts will be shaped by the ability of organisms to survive and recover physiologically from extreme events. Should organisms survive and their physiological impairment be contained to within the MHW period, it can be suggested that the structure and functioning of the original ecosystem will persist following such an event. The ability of organisms to recover from stressors will depend, in part, on the magnitude of the stress experienced, and whether key physiological processes and biological functions have been permanently disrupted (e.g., molecular damage)^13,14^.

Increasing temperatures are particularly important in the tropics as organisms in these regions live closer to their upper thermal limits, lack warming tolerance, and often have lower acclimation capacity compared to those in temperate regions^15,16^. Ectotherms living within tropical regions may be particularly susceptible relative to their temperate counterparts as changes in temperature alter the speed of their biochemical reactions and rate of metabolic processes, potentially surpassing their tolerance limits, which then scale up to alter physiological, behavioural, and ecosystem processes^1,5^. Ectotherms of particular ecological significance include marine gastropods, largely due to their role in herbivory and maintenance of algal populations^17,18^. Herbivore survival and performance under future climates have been linked with the likelihood of changes in dominant species, ecosystem alterations, and community phase shifts^19,20^.

The performance of gastropods will depend on both behavioural and physiological responses to environmental conditions. In terms of behaviour, self-righting is common in benthic invertebrates and is important for survival (i.e., predator escape, foraging) and can be used as a proxy for general neuromuscular functioning^21,22^. In terms of physiology, aerobic metabolic rates, which can be inferred from rates of oxygen consumption, indicate the energy used in physiological processes^23,24^. While changes in both behaviour and physiology are likely with changes in temperature, de-coupling of these traits has been shown in species susceptible to large environmental fluctuations^25^ as it allows their survival and functioning when exposed to adverse conditions. Thus, understanding how MHWs will alter the biological functioning of marine gastropods will be central to forecasting future ecosystem structure and function.

To investigate how MHW magnitude can affect organism persistence and performance, we conducted a laboratory investigation using the common tropical gastropod grazer, *Lunella granulata*. Survival, behavioural traits, and physiological functioning were assessed both during and after the MHW events of different magnitudes to consider the impacts of stress on these organisms, and their recovery capacity following such events. By providing an increased understanding of how MHW magnitude may drive effects on organism persistence and performance, especially if effects continue after completion of the MHW event, results will provide insights into the potential for such events to drive shifts in organisms that could ultimately modify the structure and functioning of entire ecosystems.

## Results

### Survival

All individuals survived through both the heatwave period—across all temperatures considered—and the recovery period to the end of the experimental exposure.

### Behaviour

#### Emergence

**Figure 1:**
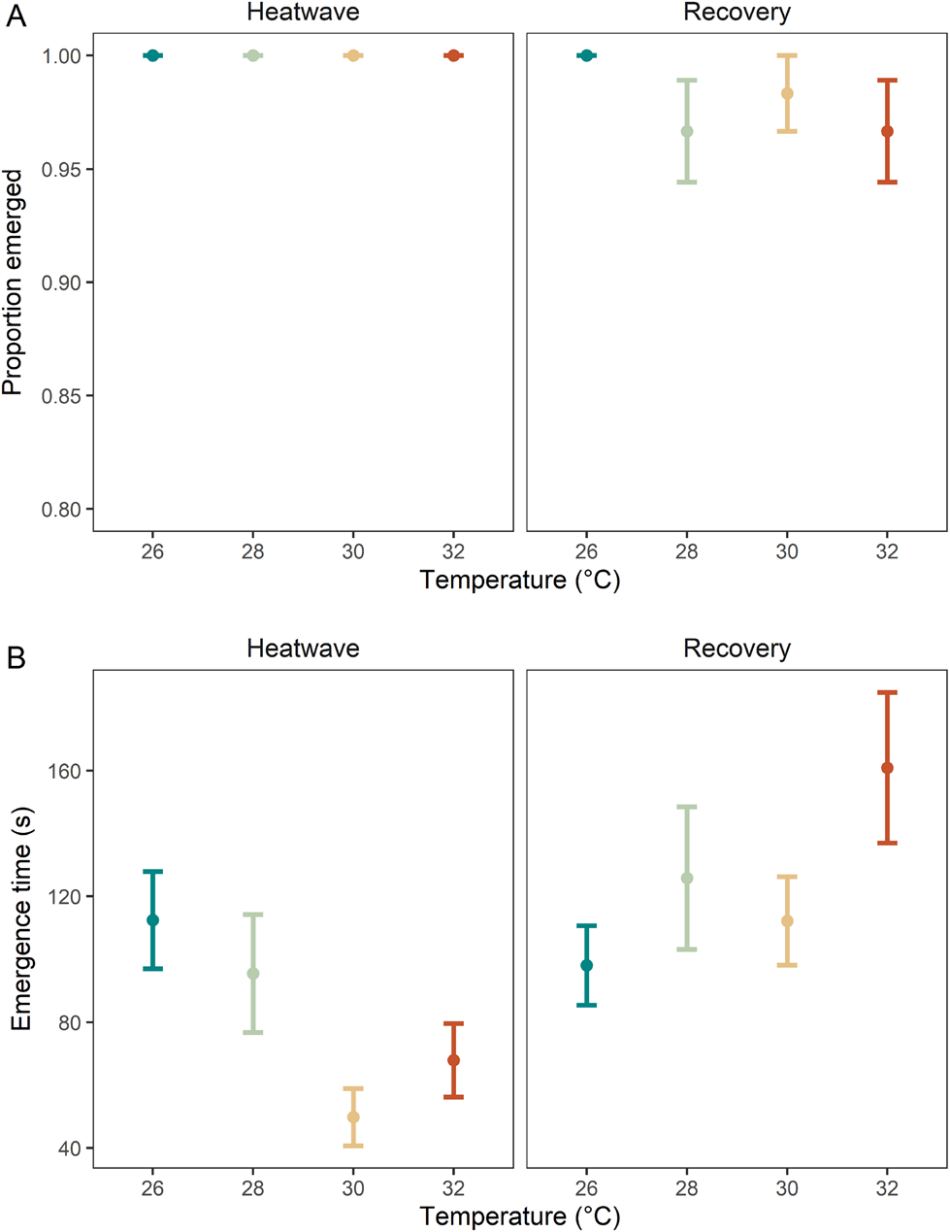
(**A**) Proportion of individuals emerged from shell during heatwave (left) and recovery (right), and (**B**) time taken for individuals to emerge from shells during heatwave (left) and recovery (right). All values are mean per replicate tank ± SE.

The majority of individuals emerged across all MHW treatments for the heatwave and recovery period, with > 99% emerging within the observation time used, and the proportions to complete this behaviour were similar among MHW magnitudes and experimental periods (Fig. 1A, Table 1A). The time taken to emerge, however, was affected by the magnitude of MHW experienced (i.e., temperature), with the response modified by experimental period (Fig. 1B, Table 1A; *p* < 0.001). During the heatwave period, emergence time decreased with increasing temperature from 26 °C to 28 °C and 30 °C, however, further increases in temperature to 32 °C induced increased emergence time (Fig. 1B). Post-hoc analysis revealed that the only significant difference in emergence time during the heatwave period was the reduction between 26 °C and 30 °C (*p* < 0.05, Supplementary Table S1A). During the recovery period, however, emergence time tended to show the opposite trend; emergence time increased with greater heatwave temperatures although there were no significant differences. In comparing the two experimental time periods, both the 30 °C and 32 °C MHWs led to significantly lower emergence times in the heatwave period compared to the recovery period, but there were no differences across time periods for the control (i.e., 26 °C) or the moderate (28 °C) treatment (Supplementary Table S1A).

**Table 1:** Statistical models examining the effects of Temperature (MHW magnitude) and Period (heatwave or recovery) on the proportion of individuals to perform (generalised linear mixed-effects model) and time taken to (cox mixed-effects model): (A) emerge from shell, (B) attempt to right, and (C) successfully right. Bold text indicates statistical significance of terms.

	Proportion			Time		
	Df	χ^2^	*p*	Df	χ^2^	*p*
**(A) Emergence**						
Temperature	3	0.396	0.941	3	2.142	0.544
Period	1	0.000	0.998	1	56.709	**< 0.001**
Temperature × Period	3	0.000	1.000	3	56.579	**< 0.001**
**(B) Attempt to right**						
Temperature	3	3.488	0.322	3	7.030	0.071
Period	1	1.220	0.269	1	11.865	**< 0.001**
Temperature × Period	3	2.439	0.486	3	12.133	**0.007**
**(C) Righting success**						
Temperature	3	3.813	0.282	3	3.497	0.321
Period	1	8.171	**0.004**	1	18.381	**< 0.001**
Temperature × Period	3	2.811	0.422	3	4.874	0.181

#### Attempt to right

**Figure 2:**
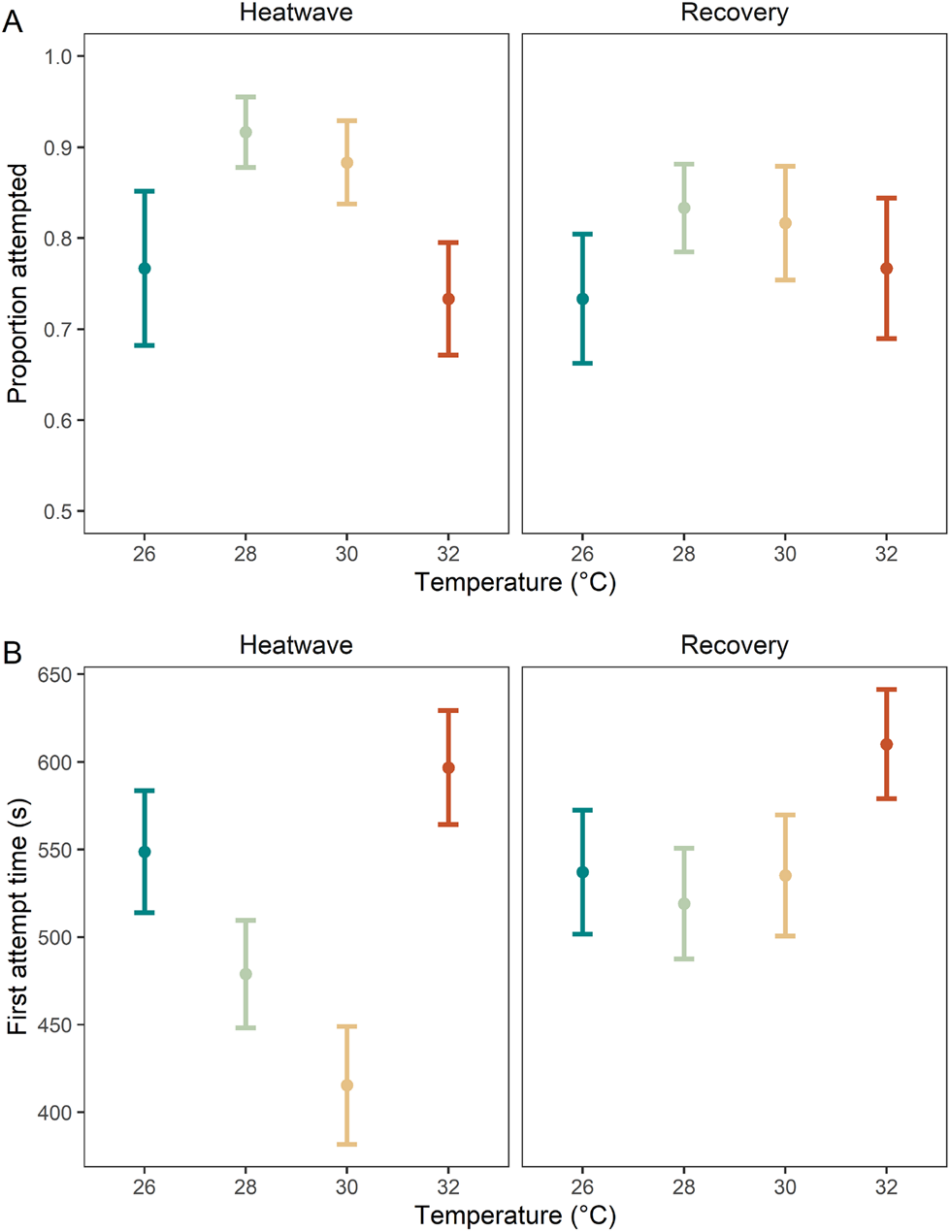
(**A**) Proportion of individuals that attempted to right during heatwave (left) and recovery (right), and (**B**) time taken for individuals to attempt to right themselves during heatwave (left) and recovery (right). All values are mean per replicate tank ± SE.

Attempt to right behaviour was observed in > 80% of individuals and was unaffected by MHW temperatures in both the heatwave and recovery periods (Fig. 2A, Table 1B). The time taken to first attempt to right was influenced by an interactive temperature and time period effect (Fig. 2B, Table 1B; *p* < 0.001). Similarly to emergence times, first attempt time decreased with increasing temperature from 26 °C to 28 °C to 30 °C during the heatwave period, however, further increases in temperature to 32 °C induced increased attempt to right times (Fig. 2B); the only significant difference was between the 30 °C and 32 °C treatments, owing to the increase in attempt to right time under the warmer temperature (*p* < 0.05, Supplementary Table S1B). In the recovery period, attempt to right time was similar across the 26 °C, 28 °C, and 30 °C treatments, however, individuals in the 32 °C MHW treatment tended to have an increased time to attempt to right although the difference was not significant. Comparing across time periods, the 30 °C treatment showed a significant difference in attempt to right time between the heatwave and recovery periods, with increased attempt to right times in the recovery compared to heatwave period (*p* < 0.05, Supplementary Table S1B).

#### Righting success

**Figure 3:**
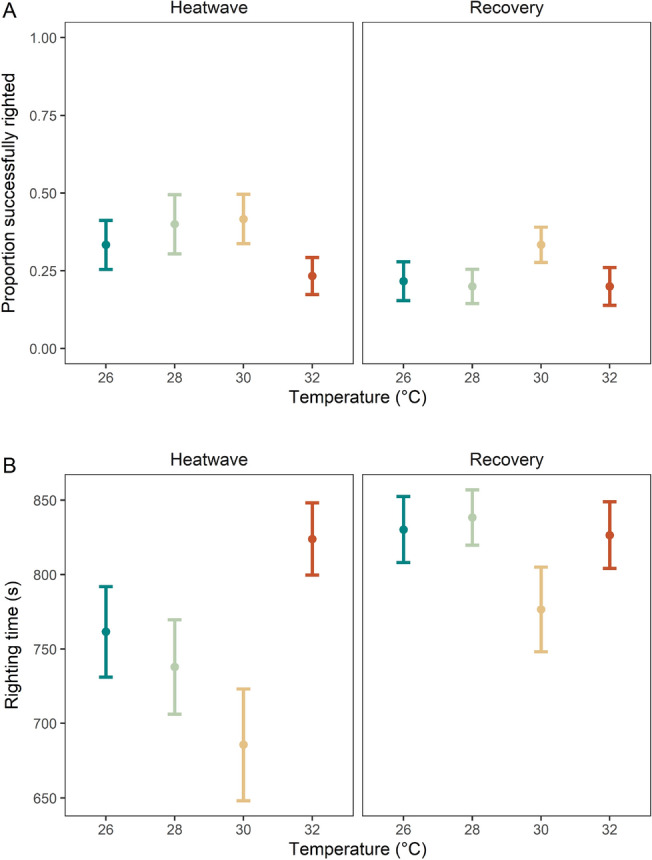
(**A**) Proportion of individuals that successfully righted themselves during heatwave (left) and recovery (right), and (**B**) time taken for individuals to successfully right themselves during heatwave (left) and recovery (right). All values are mean per replicate tank ± SE.

The proportion of individuals that successfully righted during the heatwave period was, on average, ~ 35% which was significantly greater than during the recovery period, where the proportion to successfully right was ~ 24% (*p* < 0.05, Fig. 3A, Table 1C, Supplementary Table S2). In terms of righting time, there was a significant main effect of time period (*p* < 0.05, Fig. 3B, Table 1C) with this behaviour taking less time in the heatwave than the recovery period (*p* < 0.05, Supplementary Table S1C). While the same pattern of behavioural timings was observed for righting time during the heatwave as for emergence and attempt to right (i.e., reduced righting time from 26 °C to 28 °C to 30 °C, with 32 °C being slower), the differences among treatments were not significant here (*p* > 0.05).

### Oxygen consumption

**Figure 4:**
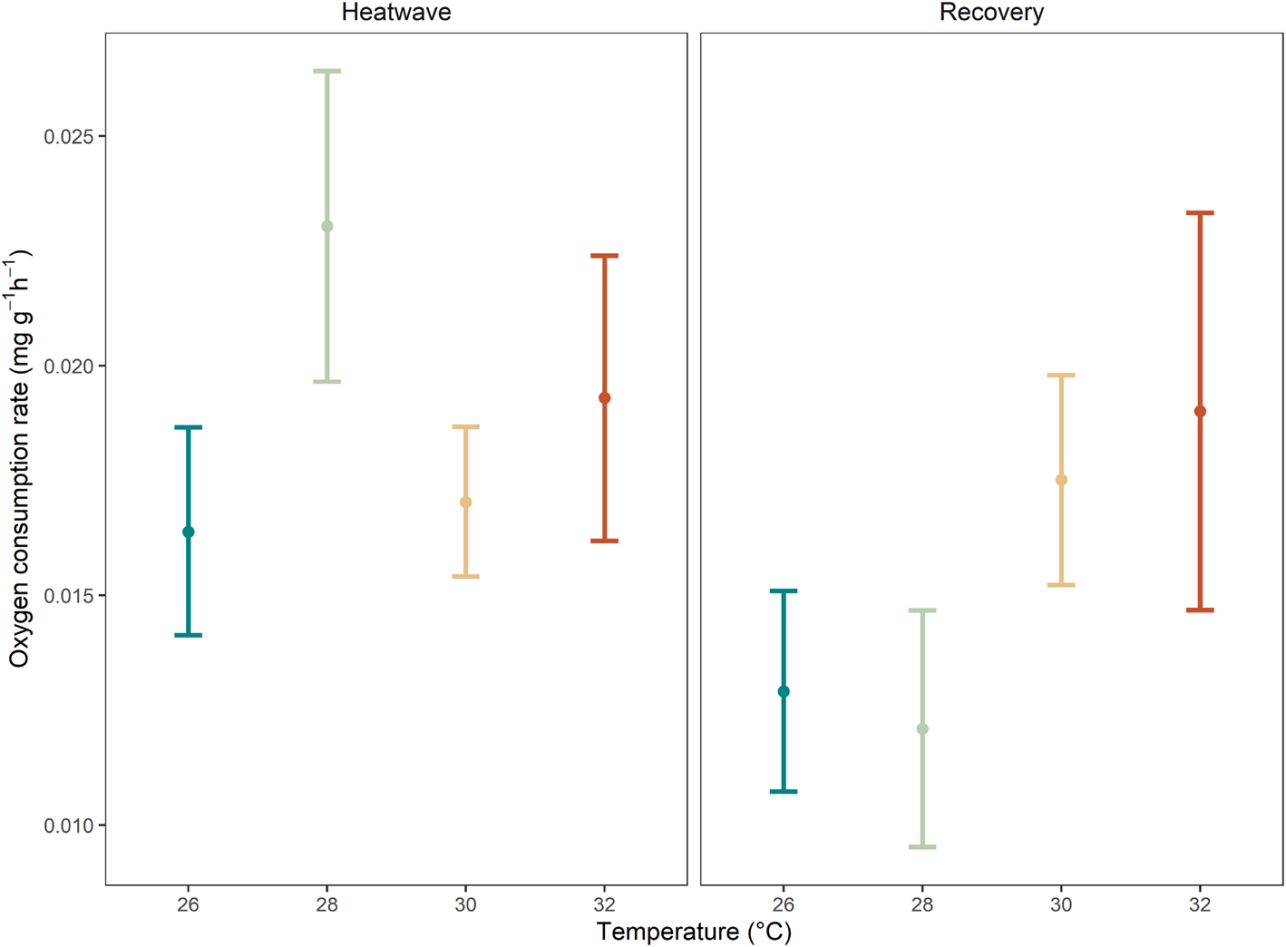
Rates of oxygen consumption for individuals during MHW (left) and recovery (right). All values are mean per replicate tank ± SE.

There was a marginally significant effect of time period (i.e., during heatwave vs recovery) on oxygen consumption rates, with higher overall rates of oxygen consumption during the heatwave compared to the recovery period (*p* < 0.05, Fig. 4, Table 2, Supplementary Table S3). No significant differences were observed for the interaction between temperature and time period, or the main effect of MHW temperature (both *p* > 0.05, Table 2).

**Table 2:** Linear mixed-effects models examining the effects of Temperature (MHW magnitude) and Period (heatwave or recovery) on oxygen consumption rates. Bold values indicate statistical significance.

	Df	χ^2^	*p*
Temperature	3	2.385	0.496
Period	1	3.917	**0.048**
Temperature × Period	3	6.294	0.098

## Discussion

Here, we show that marine heatwaves (MHWs) of varying magnitudes can alter the behavioural performance of a tropical marine gastropod, while survival and physiological functioning (i.e., oxygen consumption) remain unchanged. During moderate MHWs, the time taken to perform emergence and righting behaviours decreased, indicating they were being completed more quickly. However, extreme MHW temperatures increased the time taken to perform these same behaviours, indicating individuals were somewhat impaired. The time taken to perform righting behaviours during the recovery period by individuals exposed to moderate MHW temperatures returned to be more similar to those of the control. Those exposed to extreme MHWs, however, displayed small, but not significant, negative effects persisting into the recovery period. These behavioural changes were decoupled from survival and physiological performance, as reflected in the continued survival and maintained rates of oxygen consumption across MHWs of different magnitudes during both the heatwave and recovery periods. Consequently, our results indicate that MHWs can affect the behavioural performance of tropical gastropods, and that recovery potential may be limited after extreme events.

The magnitude of thermal change experienced during a MHW has the potential to influence the response of organisms and their ecosystem role. Here we found that under moderate MHW temperatures behaviours occurred faster (i.e., time was reduced), with these behaviours taking longer (i.e., time was increased) under the most extreme scenario considered. Such a pattern aligns with general metabolic theory which dictates that rates of organism functioning, especially in ectotherms, increase with increasing temperatures until their optimal temperature is reached, beyond which functioning begins to decrease^5^. Moreover, this result is consistent with another study of *L. granulata* in Hong Kong which identified a thermal optimum of 29.4 °C^26^; the only temperature exceeding this by > 1 °C in the current experiment prompted impaired behaviour (as indicated by the proportion to right, and time taken to right; Fig. 3). While the critical thermal maximum (CT_max_) of *L. granulata* has been found to far exceed temperatures used in our experiment (48.9 °C^26^) we show that even small increases in temperature beyond their thermal optimum can begin to induce negative behavioural effects. As the magnitude of future MHWs becomes greater under continued climate change^10^, the behaviours of these organisms and the functions they perform have the potential to be disrupted. Importantly, changes to righting behaviours as found here can provide a proxy indication of impairment of general motility, and is also related to their capacity to escape from predators and to feed^22,27^. Thus, adverse effects on righting behaviour may influence species survival in complex ecosystems (i.e., slower responses to predation risk) and disrupt ecological processes such as foraging, both of which could drive ecosystem-level effects^20,28^.

Following the end of short-term MHW events, some species are able to return behaviours to pre-event levels^13,29^, whereas others are susceptible to the latent effect of MHWs^3^. Here, during the recovery period following the moderate MHWs (28 °C and 30 °C), emergence and attempt-to-right behaviours returned to be similar to that of individuals exposed to the control temperatures (26 °C). While not statistically significant, extreme MHWs (32 °C) appeared to induce continued small negative effects after the MHW had passed. Although righting time did not show such potential for ongoing disruption of behaviour in the recovery period, this may be an artefact of the procedure used rather than the organism response. That is, the observed response may be a consequence of providing individuals that did not perform the activity within a set length of time with a censored value (15 min). Even with this approach, however, we were able to detect differences in the time taken to undertake the other two behaviours of emergence and attempt to right. Continued sublethal negative effects on organisms suggest that MHWs may be able to induce disproportionately large effects on ecosystems relative to the time during which they occur^30^.

While behavioural effects of MHW magnitude were apparent, survival and oxygen consumption rates were largely unaffected by MHW magnitude both in the heatwave and recovery periods, indicating the likelihood of continued species survival and physiological functioning after short-term events such as those simulated here. The lack of change in these traits under different temperatures may reflect an adaptation of our experimental animals to a fluctuating thermal environment, as previously found for other mollusc species^2,23^. Further, decoupling of behaviour-physiology thermal performance is a recognised response of organisms to stress (e.g.,^25^). Recognising such decoupling across behavioural and physiological performance is key to accurately predicting responses of ectothermic organisms to changes in climate^31^, and their role in future ecosystems.

With MHWs forecasted to become more intense, frequent, and longer with future climate change^9,10^, scenarios such as those simulated here will become more common for coastal organisms. Further, we suggest that the performance of future organisms could see greater impacts than observed in the current study as scenarios are exacerbated. That is, in terms of the intensity or magnitude of MHWs, we used controls at the ambient temperature when organisms were sampled (June, 26 °C). Temperatures near the collection site during the warmest month of the year (September) are on average 27 °C in bottom waters, 28.7 °C in surface waters, and can reach > 30 °C (Environmental Protection Department, Hong Kong; https://cd.epic.epd.gov.hk/EPICRIVER/marine/). If a MHW were to occur during these warmer months, we can expect that effects on organisms will be even greater, as seen for other gastropod species in Hong Kong^14^. Moreover, in the future organisms will be more likely to experience not just a single MHW as simulated here, but rather numerous events in succession. Repeated exposure to MHWs can limit the detrimental responses to subsequent events through thermal priming, however, such a pattern of exposure can also induce additive or synergistic effects leading to greater negative impacts, with the type of effect species-dependent^32–34^. Finally, MHWs are anticipated to become longer than considered here, which was the minimum duration for a MHW as defined in^8^. Thus, there is potential that extended MHWs may have greater effects on organism survival, behaviour, and physiology. Therefore, by considering a moderate MHW scenario, we are providing a conservative estimate of the effects such events could have in the future. Further, MHWs are unlikely to impact species and communities in isolation from other physical stressors. Currently, the impacts caused by MHWs are often investigated in isolation thus future studies should make efforts to quantify responses of organisms to multiple stressors.

While large-scale mortality events driven by MHWs are an obvious consequence of these events, they can also cause sublethal effects on behavioural responses and physiological functioning. Here we show that short-term increases in temperature can induce negative behavioural responses in a tropical gastropod. Such changes have the potential to influence the organism response which can, in turn, influence species interactions (specifically herbivory). Thus, understanding responses of individuals to short-term environmental fluctuations will be important in understanding how effects can scale up to impacts on ecosystem structure and functioning.

## Methods

### Organism collection and maintenance

Gastropods, *Lunella granulata*, were collected in May 2021 from the low intertidal on a rocky shore in Hong Kong (Nai Chung; 22° 26′ 0.312″ N, 114° 15′ 18.6804″ E) and returned to the Simon F.S. Li Marine Science Laboratory, The Chinese University of Hong Kong. Gastropods were maintained for 10 days in ~ 22 L tanks filled with constantly aerated, sand-filtered seawater pumped from the adjacent Tolo Harbour and held at 26 °C (i.e., ambient water temperature). After acclimation to laboratory conditions, 5 individual *L. granulata* were transferred to each of 48 replicate ~ 5 L tanks (*n* = 12 tanks per experimental group; *N* = 48 experimental tanks total) and maintained at the same conditions as the acclimation period for another 4 days (i.e., 14 days laboratory acclimation total). During the acclimation period, gastropods were cleaned of epibionts and marked with nail polish to identify individuals. Animals used in the experiment were 2.27 ± 0.03 g (mean ± s.e.). Water changes (~ 50% of total volume) were conducted every one to three days during the acclimation and experimental periods. Each experimental tank was fitted with a 50-W bar heater allowing independent replication of temperature conditions. Water temperature and salinity were monitored using a digital thermometer (Hanna Instruments, USA) and refractometer (Wiggens GmbH, Germany), respectively (Supplementary Table S4). During the acclimation and experimental periods, gastropods were fed defrosted *Ulva* sp. which had previously been collected from a nearby site, with feeding stopped three days prior to measurement of response variables.

### Marine heatwave scenarios and experimental treatments

To examine the effects of MHW magnitude on the survival, behaviour, and physiological responses of *L. granulata*, we experimentally simulated MHWs of various magnitudes (control, + 2 °C, + 4 °C, + 6 °C) for 5 days followed by 5 days of recovery with water temperatures returning to pre-MHW levels. To implement the MHW treatments, water temperatures were maintained or increased from the ambient (26 °C) to the experimental MHW temperature (26, 28, 30, or 32 °C) at ~ 1 °C h^−1^. These MHW temperatures were then maintained for 5 days, after which survival, behavioural responses, and oxygen consumption were measured at the appropriate MHW temperature. The day after measuring behavioural responses and rates of oxygen consumption (see below), water temperatures were reduced to control temperatures (26 °C) at a rate of ~ 1 °C h^−1^ and maintained for a further 5 day recovery period, after which survival, behavioural responses, and oxygen consumption were re-measured.

### Response variables

Experimental animals were monitored daily to identify any mortality throughout the MHW exposure and recovery periods.

To measure behavioural performance, all individuals were removed from the experimental tanks and placed, fully-retracted and inverted, into the centre of a separate 150 ml container filled with ~ 100 ml of source water (i.e., from the corresponding experimental tank). Once placed into the measurement container, righting behaviour was recorded for 15 min. When the 15 min recording limit was reached, animals were returned to their experimental tanks. Recordings of righting behaviour were reviewed to identify the proportion of individuals, and the time taken (s) for each individual, to: (i) emerge from its shell; (ii) first attempt to right itself; and (iii) successfully right itself. All video recordings were reviewed by one person to reduce variation in behavioural classifications. Emergence was defined as when a gap between the shell and the operculum of the snail was observed from the top view. The first attempt to right was defined as the first occurrence when an individual stretched its foot to touch the substrate. Successful righting was when the foot of the gastropod successfully attached to the substrate and the body and shell were pulled back to the original orientation^35^.

To measure oxygen consumption, two individuals per replicate tank were transferred into 50 ml airtight chambers filled with water from the experimental tanks, ensuring the absence of any gas bubbles. Each chamber was fitted with a precalibrated oxygen spot (PreSens) attached to the inner wall. Following transfer, gastropods were allowed to rest for 10 min, after which time oxygen concentrations were measured every 5 min for 20 min using a fibre optic meter (Fibox 4 Trace, PreSens). Paired blank chambers (i.e., containing no animal) were used alongside chambers containing experimental organisms for each experimental tank to account for any biological activity within the water. The oxygen consumption rate was calculated as:

$$Oxygen \, consumption \, rate \left(\mathrm{mg} {\mathrm{g}}^{-1}{\mathrm{h}}^{-1}\right) = \frac{{\Delta O}_{2}\times V}{W\times t},$$

where *ΔO*_*2*_ is the change in oxygen concentration within the chamber, *V* is the volume of water in the chamber (L), *W* is the fresh weight of the individual (g), and *t* is the time between the first and last oxygen measurements (h).

### Statistical analyses

All statistical analyses were conducted in R v4.0.2^36^. For the analysis of behavioural responses, as behavioural trials were limited to 15 min, individuals that performed or did not perform the behaviours were dichotomously scored with success or fail (1 or 0). The proportion of individuals performing the behaviours of emergence, attempt to right, and successful righting was analysed using generalised linear mixed effects models with a binomial error distribution^37^, using the fixed factors temperature (MHW magnitude) and time period (during MHW or recovery), with each replicate tank included as a random factor to account for multiple measurements from the same tank. For analysis of time taken to perform a behaviour, due to the time limitation on behavioural trials, individuals that did not perform the behaviour were censored (i.e., allocated a time of 15 min). Owing to this, we used time-to-event analysis (also known as survival analysis). A mixed-effects Cox proportional hazard model^38^ based on Kaplan–Meier estimations^39^ was used to assess the effect of MHW magnitude and time period on emergence time, attempt time, and righting success time. Temperature (MHW magnitude) and time period (during MHW or recovery) were included as fixed factors, and each replicate tank included as a random factor to account for repeated measures from each time period.

To assess the effects of temperature (MHW magnitude) and time period (during MHW or recovery) on oxygen consumption, we used a linear mixed-effects model with temperature (MHW magnitude) and time period (during MHW or recovery) included as fixed factors, and tank included as a random factor to account for repeated measures during each time period.

Statistical significance of fixed factors from all models was assessed using analysis of deviance^40^, and where significant differences occurred, pairwise comparisons were performed using estimated marginal means^41^.

## Supplementary Information

Supplementary Information.

## Data Availability

The datasets used and/or analysed during the current study available from the corresponding author on reasonable request.
