# Supplementary material for: Marine heatwaves of different magnitudes have contrasting effects on herbivore behaviour
Source: Sci Rep. 2022 Oct 15;12:17309. doi: 10.1038/s41598-022-21567-9 (PMC9569385; doi:10.1038/s41598-022-21567-9)
Supplement: Supplementary file 1 — Supplementary Information. [file 41598_2022_21567_MOESM1_ESM.docx]

Marine heatwaves of different magnitudes have contrasting effects on herbivore behaviour

Running head: Effects of marine heatwave magnitude

Patrick W.S. Joyce, Wing Yee Tang, Laura J. Falkenberg*

Simon F.S. Li Marine Science Laboratory, The Chinese University of Hong Kong, Shatin, New Territories, Hong Kong SAR

*corresponding author email: laurafalkenberg@cuhk.edu.hk

ORCiD

Patrick W.S. Joyce (0000-0003-1058-7901)

Wing Yee Tang (0000-0002-7105-8803)

Laura J. Falkenberg (0000-0002-5868-2310)

Supplementary Table S1. Significant posthoc results from cox-mixed effects models examining the effects of temperature (MHW magnitude) and period (heatwave or recovery) on the time taken to: A) emerge from shell, B) attempt to right, and C) successfully right.

| Contrast | Estimate | SE | z.ratio | *p* |
| --- | --- | --- | --- | --- |
| *A) Emergence* |  |  |  |  |
| 26 Heatwave - 30 Heatwave | -1.2497 | 0.356 | -3.509 | 0.0106 |
| 28 Heatwave - 32 Recovery | 1.0999 | 0.36 | 3.052 | 0.0469 |
| 30 Heatwave - 28 Recovery | 1.521 | 0.359 | 4.24 | 0.0006 |
| 30 Heatwave - 30 Recovery | 1.6031 | 0.232 | 6.918 | <.0001 |
| 30 Heatwave - 32 Recovery | 2.1364 | 0.366 | 5.832 | <.0001 |
| 32 Heatwave - 28 Recovery | 1.1952 | 0.36 | 3.321 | 0.0202 |
| 32 Heatwave - 30 Recovery | 1.2773 | 0.357 | 3.577 | 0.0083 |
| 32 Heatwave - 32 Recovery | 1.8106 | 0.223 | 8.124 | <.0001 |
| *B) Attempt to right* |  |  |  |  |
| 30 Heatwave - 32 Heatwave | 1.4806 | 0.458 | 3.231 | 0.0271 |
| 30 Heatwave - 30 Recovery | 1.0449 | 0.235 | 4.453 | 0.0002 |
| 30 Heatwave - 32 Recovery | 1.6844 | 0.459 | 3.667 | 0.006 |
| *C) Righting success* |  |  |  |  |
| Heatwave - Recovery | 0.766 | 0.186 | 4.109 | <.0001 |

Supplementary Table S2. Significant posthoc results from generalised linear model examining the effects of period (heatwave or recovery) on the proportion of individuals able to successfully right.

| Contrast | Estimate | SE | z.ratio | *p* |
| --- | --- | --- | --- | --- |
| Heatwave - Recovery | 0.75 | 0.255 | 2.94 | 0.0033 |

Supplementary Table S3. Significant posthoc results from generalised linear model examining the effects of period (heatwave or recovery) on the oxygen consumption rates of *Lunella granulata*.

| Contrast | Estimate | SE | z.ratio | *p* |
| --- | --- | --- | --- | --- |
| Heatwave - Recovery | 0.00356 | 0.0018 | 1.979 | 0.0508 |

Supplementary Table S4. Abiotic water parameters measured during the heatwave and recovery periods for each MHW treatment (i.e., 26, 28, 30, and 32°C). All parameters were measured for *n* = 5 days per time period (i.e., heatwave or recovery).

|  | Temperature (°C) | | | | | | Salinity | | | | | |
| --- | --- | --- | --- | --- | --- | --- | --- | --- | --- | --- | --- | --- |
|  | Heatwave | | | Recovery | | | Heatwave | | | Recovery | | |
| MHW treatment | Mean | SE | Mean | | SE | Mean | | SE | Mean | | SE |  |
| 26 | 26.0 | 0.1 | 25.7 | | 0.1 | 35 | | <1 | 35 | | <1 |  |
| 28 | 28.1 | <0.1 | 25.8 | | 0.1 | 35 | | <1 | 35 | | <1 |  |
| 30 | 29.9 | 0.1 | 25.7 | | 0.1 | 36 | | <1 | 36 | | <1 |  |
| 32 | 32.0 | 0.1 | 25.7 | | 0.1 | 36 | | <1 | 36 | | <1 |  |
